# SARS‐CoV‐2 spike protein harnesses SNX27‐mediated endocytic recycling pathway

**DOI:** 10.1002/mco2.92

**Published:** 2021-10-08

**Authors:** Lin Zhao, Kunhong Zhong, Jia Zhao, Xin Yong, Aiping Tong, Da Jia

**Affiliations:** ^1^ Key Laboratory of Birth Defects and Related Diseases of Women and Children Department of Paediatrics State Key Laboratory of Biotherapy and Collaborative Innovation Center of Biotherapy West China Second University Hospital Sichuan University Chengdu China; ^2^ State Key Laboratory of Biotherapy and Cancer Center West China Hospital West China Medical School Sichuan University Chengdu China

**Keywords:** endocytic trafficking, endosome, host–pathogen interaction, S protein, SARS‐CoV‐2

## Abstract

SARS‐CoV‐2 is an enveloped positive‐sense RNA virus that depends on host factors for all stages of its life. Membrane receptor ACE2 is a well‐established factor for SARS‐CoV‐2 docking. In addition to ACE2, whole‐genome genetic screens have identified additional proteins, such as endosomal trafficking regulators SNX27 and retromer, as key host factors required for SARS‐CoV‐2 infection. However, it is poorly understood how SARS‐CoV‐2 utilize host endocytic transport pathways to produce productive infection. Here, we report that SNX27 interacts with the SARS‐CoV‐2 spike (S) protein to facilitate S protein surface expression. Interestingly, S protein binds to the PDZ domain of SNX27, although it does not contain a PDZ‐binding motif (PDZbm). Either abrogation of the SNX27 PDZ domain or S protein “MTSC” motif, which is critical for SNX27 binding, decreases surface expression of S protein and viral production. Collectively, our study highlights a novel approach utilized by SARS‐CoV‐2 to facilitate virion trafficking to establish virus infection.

## Graphical abstract

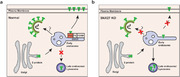

## 1 INTRODUCTION

As one of the most notorious infectious diseases the modern human history, the novel coronavirus 2019 (COVID‐19) has infected at least 200 million individuals, as of August 31, 2021, and led to a global health and socioeconomic crisis (https://coronavirus.jhu.edu/map.html). The causative agent of COVID‐19, SARS‐CoV‐2 causes a respiratory tract infection accompanied by a mild fever, which can progress to severe acute respiratory syndrome, multi‐organ dysfunction, and even death within a short period.^1^, ^2^, ^3^ SARS‐CoV‐2 enters host cells through two distinct pathways: fusion at the plasma membrane or endocytosis.^4^ Both pathways rely on the spike protein (referred to as S protein) of SARS‐CoV‐2 binding to ACE2 at cell surface.^5^, ^6^, ^7^, ^8^, ^9^ The presence of furin protease, trypsin and TMPRSS2 in host cells, leads to cleavage at the S1/S2 junction site and additional sites of S protein, and promotes fusion at the plasma membrane. Otherwise, SARS‐CoV‐2 will be endocytosed.^10^, ^11^, ^12^

Membrane receptors, together with their bound protein or lipids, enter the cells via endocytosis. Some of them could be recycled back to the plasma membrane, a process often depend on retromer, retriever, or members of the sorting nexin (SNX) family.^13^, ^14^, ^15^, ^16^ As a member of the SNX family, SNX27 plays a critical role in endosomal receptor recycling, often together with retromer and SNX‐BARs.^14^, ^17^, ^18^ SNX27 harbors a PDZ domain, which can bind to the PDZ‐binding –motif (PDZbm) presented in the cytoplasmic tails of many membrane proteins, including the glucose transporter GLUT1 and many G‐protein–coupled receptors, and promotes their endosome‐to‐plasma membrane recycling.^14^, ^19^ In addition to angiotensin‐converting enzyme 2 (ACE2), the surface receptor for S protein, SARS‐CoV‐2 infection requires dozens of host factors. Whole genome CRISPR screens have identified that endocytic trafficking regulators, such as SNX27 and retromer, are required for SARS‐CoV‐2 infection.^20^, ^21^ Interestingly, a separate study shows that SNX27 interacts with the SARS‐CoV‐2 S protein.^22^ S protein contains a short cytoplasmic tail, in addition to a large external domain and a transmembrane domain.^23^ However, it does not encompass a PDZbm. Therefore, the biological significance of the SNX27‐S interaction is unclear, and it remains largely unknown how SNX27 facilitates SARS‐CoV‐2 infection.

To determine the functions of SNX27 in SARS‐CoV‐2 infection, we investigated how SNX27 influenced the transfection efficacy of S‐bearing pseudovirus. We found that SNX27 mediates endocytic trafficking of S protein, promotes its surface expression, and enhances the transfection efficacy of S‐bearing pseudovirus. Interestingly, the PDZ domain of SNX27 is critical for the interaction with S protein, although S protein does not encompass a PDZbm. Our study indicates that SNX27 may facilitate SARS‐CoV‐2 infection via promoting endocytic trafficking of S protein.

## 2 RESULT

### 2.1 SNX27 promotes transduction efficiency of S‐bearing pseudovirus

**FIGURE 1:**
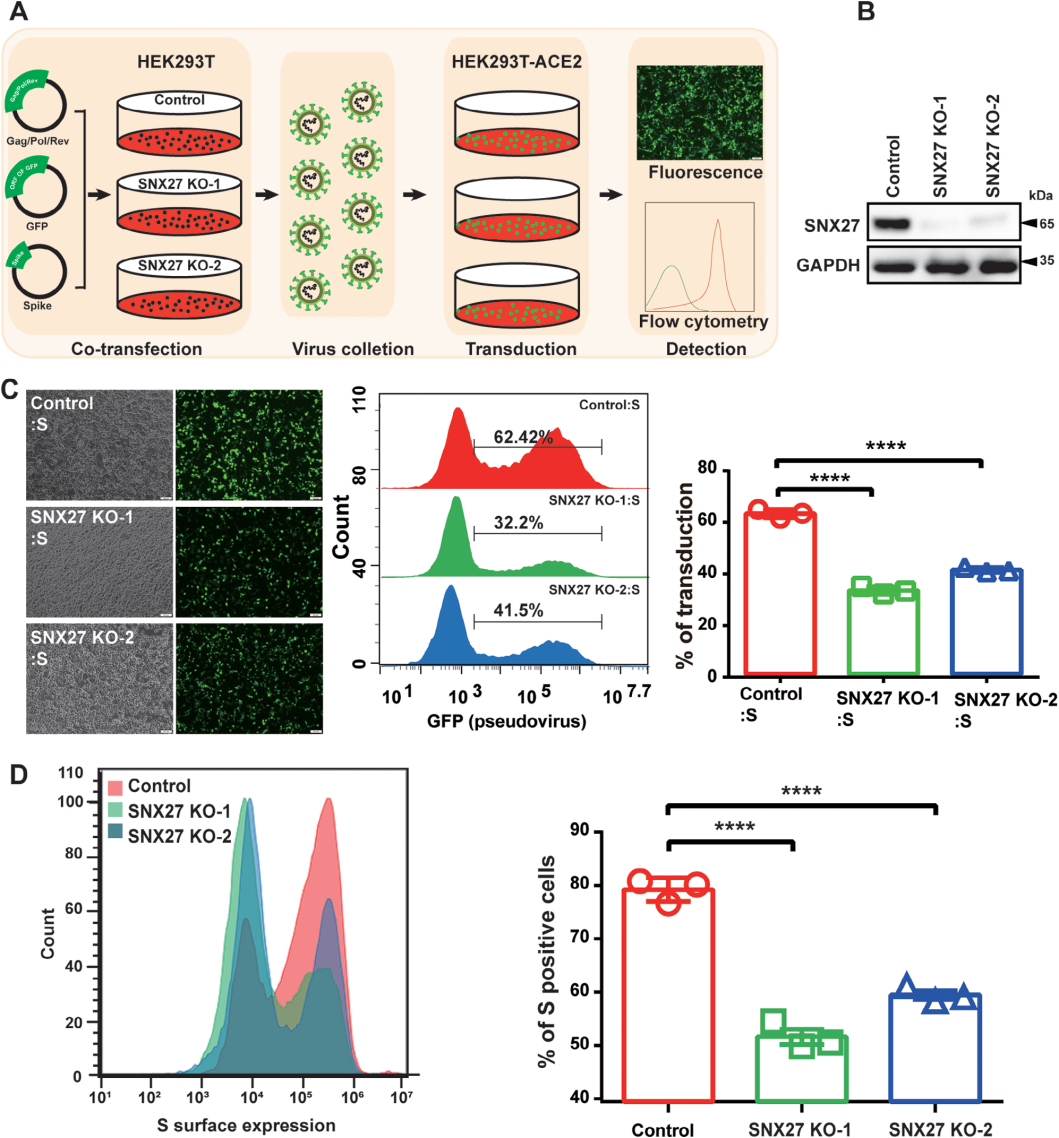
SNX27 facilitates transduction efficiency of the S‐bearing pseudovirus and cell surface expression of S protein. (A) Workflow of the S‐bearing pseudovirus transduction efficiency analysis. SARS‐CoV‐2 S‐bearing pseudovirus was produced in WT or SNX27 KO HEK293T cells. Next, the virus‐containing supernatant was harvested and used to transduce ACE2‐expressing HEK293T cells (HEK293T‐ACE2). (B) Immunoblotting analysis of SNX27 protein expression in HEK293T cells transfected with CRISPR‐Cas9 plasmids targeting SNX27 (KO‐1 and KO‐2 employing two different gRNAs), or empty vector (control). (C) Fluorescence and flow cytometry analysis of transduction efficiency of the S‐bearing pseudoviruses produced from cells in b. Left: representative fluorescence images from one experiment. Middle: representative flow cytometry histograms from one experiment. Right: the percent of GFP positive cells, determined by flow cytometry, is used to calculate viral transduction efficiency. Data are mean ±SD of three independent experiments. ****p < 0.0001. Scale bar: 100 µm. (D) Determination of S protein cell surface expression by flow cytometry. HEK293T cells were transiently transfected with plasmids encoding full‐length S. Twenty‐four hours after transduction, cells were collected and stained with anti‐S antibody (which recognizes extracellular domain of S protein), followed by flow cytometer analysis. Left: flow cytometry results from one representative experiment. Right: percent of S‐positive cells. Statistic data represent the results from *n* = 3 independent experiments and are expressed as mean ± SD. ****p < 0.0001. Scale bar: 100 µm

SNX27 is reported to mediate the trafficking of ACE2, a host receptor essential for SARS‐CoV‐2 infection.^20^, ^24^ To precisely define the function of SNX27 in regulating S protein and to distinguish its role in regulating ACE2, we developed a procedure, as illustrated in Figure 1A. First, SARS‐CoV‐2 S‐bearing pseudovirus was produced in WT or SNX27 knockout (KO) HEK293T cells. SNX27 KO cells were generated using CRISPR/Cas9‐mediated gene editing technology, and knockdown efficiency reached approximately 90% as determined by immunoblotting (Figure 1B). Second, the virus‐containing supernatant was harvested, and used to transduce ACE2‐expressing HEK293T cells (HEK293T‐ACE2). Both fluorescence and flow cytometry assay revealed that suppression of SNX27 significantly decreased in the transduction efficiency of S‐bearing pseudovirus (Figure 1C). Approximately 32.2 % and 41.5% of HEK293T‐ACE2 cells were infected by S‐bearing pseudovirus produced from SNX27 KO‐1 and KO‐2 cells, in comparison with 64.4% for control cells (Figure 1C).

One major difference between the SARS‐CoV‐2 S protein and the SARS‐CoV S protein is the insertion of furin‐cleavage site (FCS, _682_‐RRAR/AAAA‐_685_). To test whether FCS is required for the regulation of S protein by SNX27, we tested the transduction efficiency of the S‐FKO‐bearing pseudovirus (FKO: deletion of furin‐cleavage site; Figure S1A). Similar to S‐bearing pseudovirus, depletion of SNX27 also reduced the transduction efficiency of S‐FKO‐bearing pseudovirus, indicating that SNX27 regulates the function of S protein independent of FCS. To further dissect the role of SNX27 in regulating S protein, we transfected plasmids encoding S or S‐FKO protein into control, SNX27 KO‐1 and KO‐2 HEK293T cells, respectively. The cells were then collected for flow cytometry analysis. Loss of SNX27 led to reduced membrane expression of both S (Figure 1D) and S‐FKO (Figure S1B). Thus, SNX27 may help SARS‐CoV‐2 infection by promoting the surface expression of S protein.

### 2.2 SNX27 mediates intracellular transport of S protein

**FIGURE 2:**
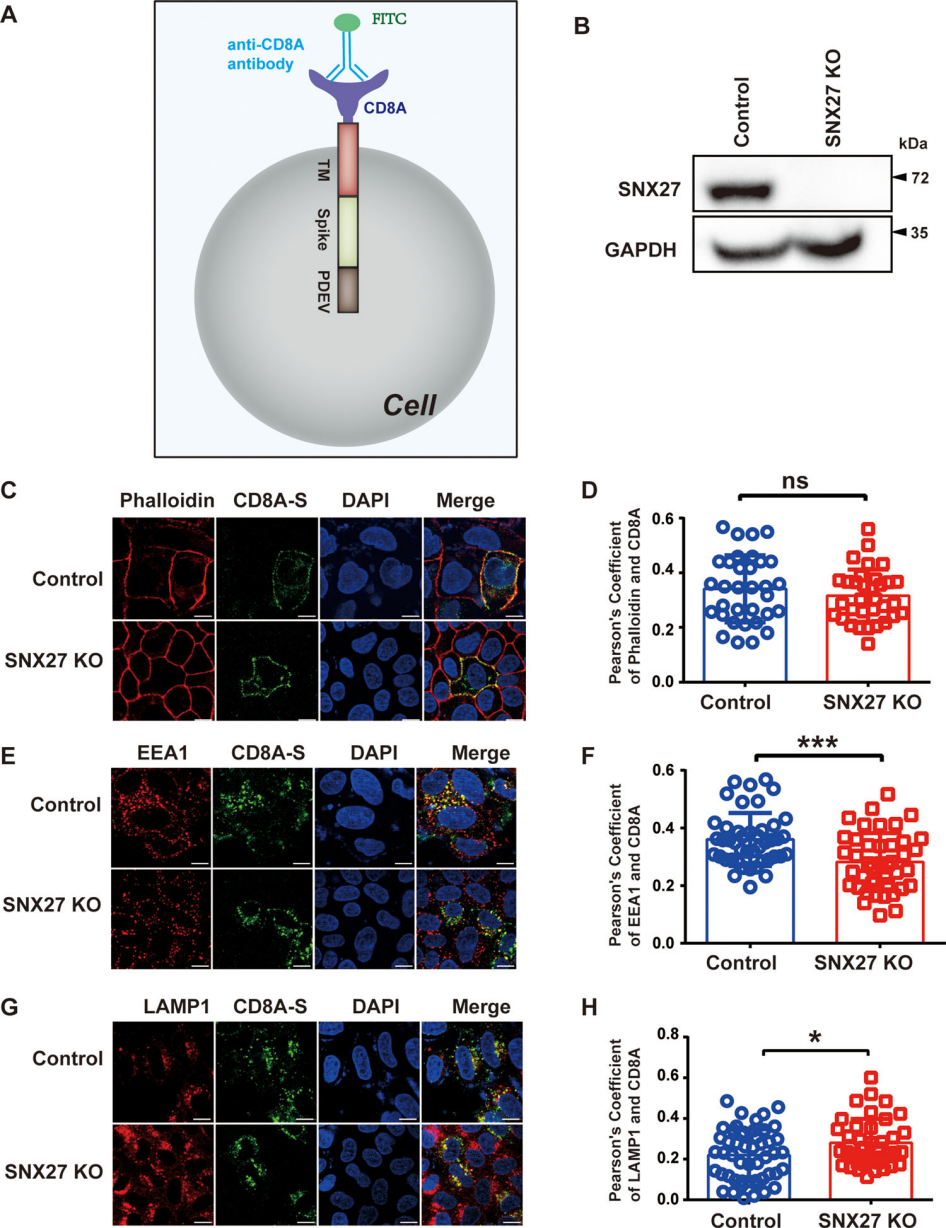
SNX27 promotes endocytic recycling of S protein. (A) A cartoon of CD8A‐S chimera used in the experiment. The CD8A‐S chimera construct consists of CD8A (1–205 aa), a linker peptide, S protein (1235–1256 aa), and the PEDV S protein (1374–1387 aa). The PEDV S protein contains an adaptor protein complex 2 (AP‐2) recognition motif, which is used to facilitate endocytosis. (B) Depletion efficiency of SNX27 in HeLa cells, determined by immunoblotting. (C)–(H) Control, SNX27‐KO HeLa cells were transiently transfected with plasmids encoding CD8A‐S for 24 h. Cells were incubated with monoclonal anti‐CD8A antibody on ice for 30 min. Unbound antibodies were removed, and the internalization of antibody‐bound CD8A was chased in DMEM at 37˚C for 0 min (C, D), 30 min (E, F), and 60 min (G, H), respectively. The internalized CD8A–antibody was detected using Alexa‐488 secondary antibodies, with cell membrane stained with phalloidin (C, D), early endosome stained with EEA1 (E, F), lysosomes stained with LAMP1 (G, H). Scale bar: 10 µm. (D) Quantification of CD8A/phalloidin colocalization in cells in C. (F) Quantification of CD8A/EEA1 colocalization in cells in E. (H) Quantification of CD8A/LAMP1 colocalization in cells in G. Each dot represents Pearson's correlation coefficients from one cell. *N* = 3 independent experiments. *p* Values were calculated using one‐way ANOVA, Tukey's multiple comparisons test. Ns: no significant difference. *p < 0.05; ***p < 0.001

SNX27 is known to mediate endosome‐to‐plasma membrane recycling of membrane proteins.^14^ To explore whether SNX27 mediates endocytic trafficking of S protein or not, we engineered a CD8A‐S chimera by fusing the extracellular region of human CD8a with the cytoplasmic tail of S protein^25^ (Figure 2A). An endocytic motif from porcine epidemic diarrhea virus (PEDV) S protein was attached to the C‐terminus of the constructs in order to enhance endocytosis.^26^ We performed anti‐CD8A antibody uptake assays in control and SNX27 KO HeLa cells^17^ (Figure 2B–H). Depletion of SNX27 did not impair the surface expression of CD8A‐S at the plasma membrane at the beginning of endocytosis, as determined by the colocalization of CD8A and phalloidin 10 min after internalization (Figures 2C and 2D). However, deletion of SNX27 resulted in decreased colocalization of CD8A‐S with EEA1, an early endosomal marker, as assessed by the colocalization of CD8A and EEA1 30 min after internalization (Figures 2E and 2F). Meanwhile, deletion of SNX27 increased colocalization of CD8A‐S with LAMP1, a late endosomal marker (Figures 2G and 2H). Taken together, these results suggest that SNX27 may help endocytic recycling of S protein and prevent it from entering the lysosomal degradation pathway.

**FIGURE 3:**
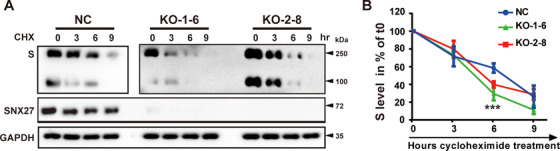
Depletion of SNX27 increases degradation of SARS‐CoV‐2 S protein. (A), (B) S protein degradation assays. Two clonal SNX27‐KO cell lines (KO‐1‐6 and KO‐2‐8) were selected from SNX27 KO‐1 and SNX27 KO‐2, respectively. Control and SNX27‐KO cells were transiently transfected with plasmid encoding S protein. Twenty‐four hours after transfection, cells were treated with cycloheximide (CHX, 25 µg/ml) for the indicated time periods. The amount of S protein was expressed relative to the amount of GAPDH (loading control) and then normalized to the sample at 0 h. Graph shows the degradation kinetics (B), with error bars indicating SD. ***p < 0.001 comparison of control and SNX27 KO in a one‐way ANOVA test. Experiments were repeated four times

To further verify the role of SNX27 in promoting endocytic trafficking of S protein, we performed protein degradation assays in the absence or presence of ribosomal inhibitor cycloheximide (CHX). These assays indicated that depletion of SNX27 enhanced S protein degradation (Figures 3A and 3B). In control cells, about 40% of S protein was degraded 6 h after CHX treatment; in both SNX27 KO clonal cells (KO‐1‐6 and KO‐2‐8) that we tested, over 60% of S protein was degraded at the same time point (Figure 3B). In addition, the lysosome inhibitor Chloroquine (CQ), but not the proteasome inhibitor MG132, partially rescued S protein degradation caused by SNX27 deletion (Figure S2). These results are consistent with a model that SNX27 promotes endocytic recycling of S protein. Depletion of SNX27 results in lysosomal trafficking of S protein and subsequent degradation.

### 2.3 The PDZ domain of SNX27 binds to S protein

**FIGURE 4:**
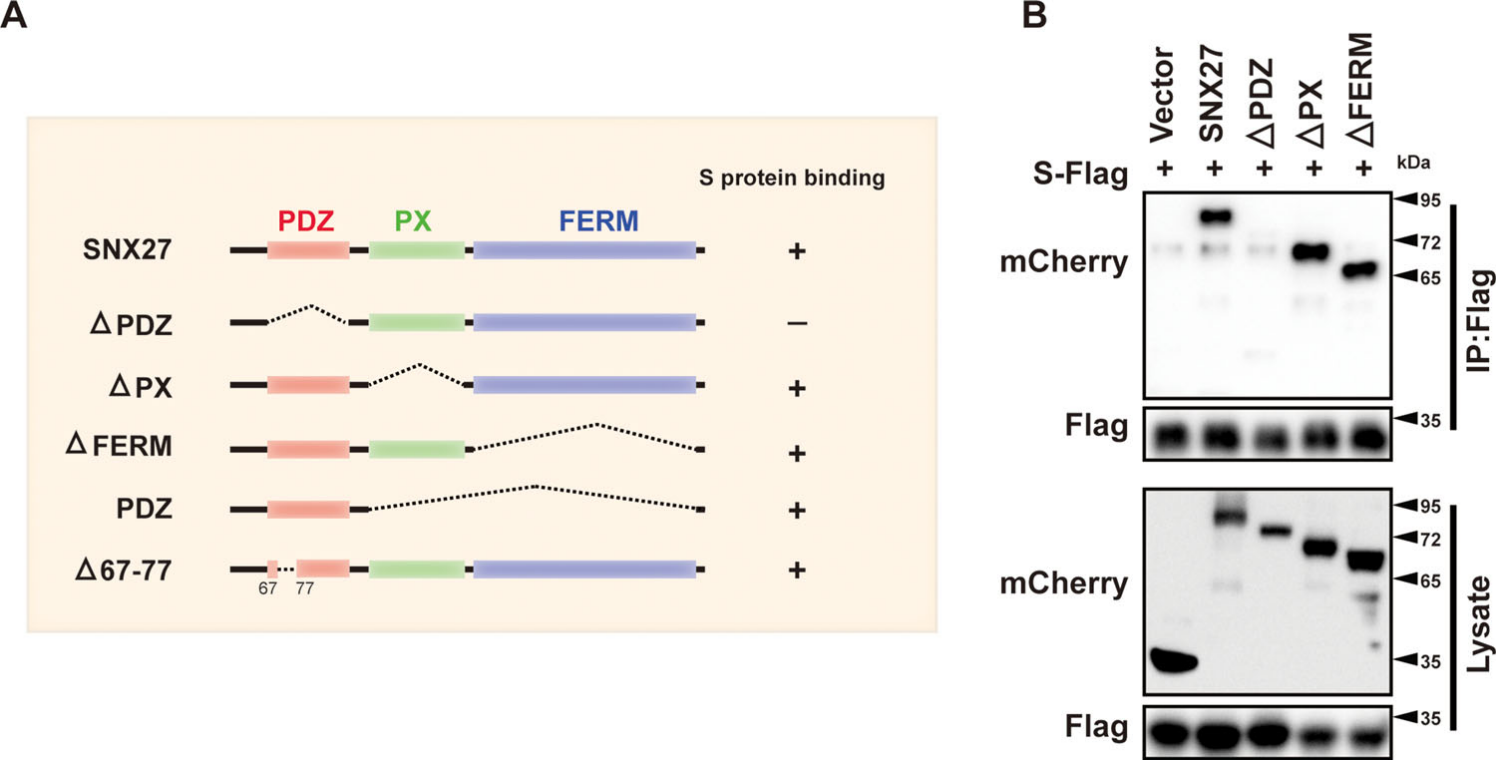
The PDZ domain of SNX27 is required for interaction with S protein. (A) Schematic representation of full‐length SNX27 and SNX27‐△PDZ, ‐△PX, ‐△FERM, ‐PDZ, and △67‐77 mutants. (B) Vector, SNX27, SNX27‐△PDZ, ‐△PX, and ‐△FERM were cotransfected with S‐Flag (GST‐S_1235‐1273_‐3×Flag) in HEK293T cells. Cells were lysed, and immunoprecipitation was performed using anti‐Flag magnetic beads. The cell lysate (bottom) and beads‐bound proteins (top) were visualized by immunoblotting

SNX27 encompasses three structural domains: PDZ, PX, and FERM domains. The PDZ domain of SNX27 can engage with both retromer and PDZbms at the C‐terminus of transmembrane proteins.^14^, ^27^, ^28^ The PX domain interacts with PI(3)P and is thought to be critical for membrane recruitment of SNX27; in contrast, the functions of FERM domain remain largely unknown. To determine which region of SNX27 binds to S protein, we generated a series of SNX27 deletion constructs and performed immunoprecipitation assays (Figure 4A). Coimmunoprecipitation revealed that deletion of the PDZ domain (△PDZ), but not PX (△PX) or FERM domain (△FERM) abolished the interaction between SNX27 and S protein, suggesting that the PDZ domain of SNX27 is required for the binding. (Figures 4A and 4B). Furthermore, we found that the PDZ domain of SNX27 (SNX27‐PDZ) was sufficient to bind to S protein (Figures 4A and S3). Interestingly, a SNX27 mutant (△67–77) deficient for retromer binding retained the ability to interact with S protein, suggesting that SNX27 associates with S protein independent of retromer (Figure S3). These results collectively demonstrate that the PDZ domain is required and sufficient for binding to S protein.

**FIGURE 5:**
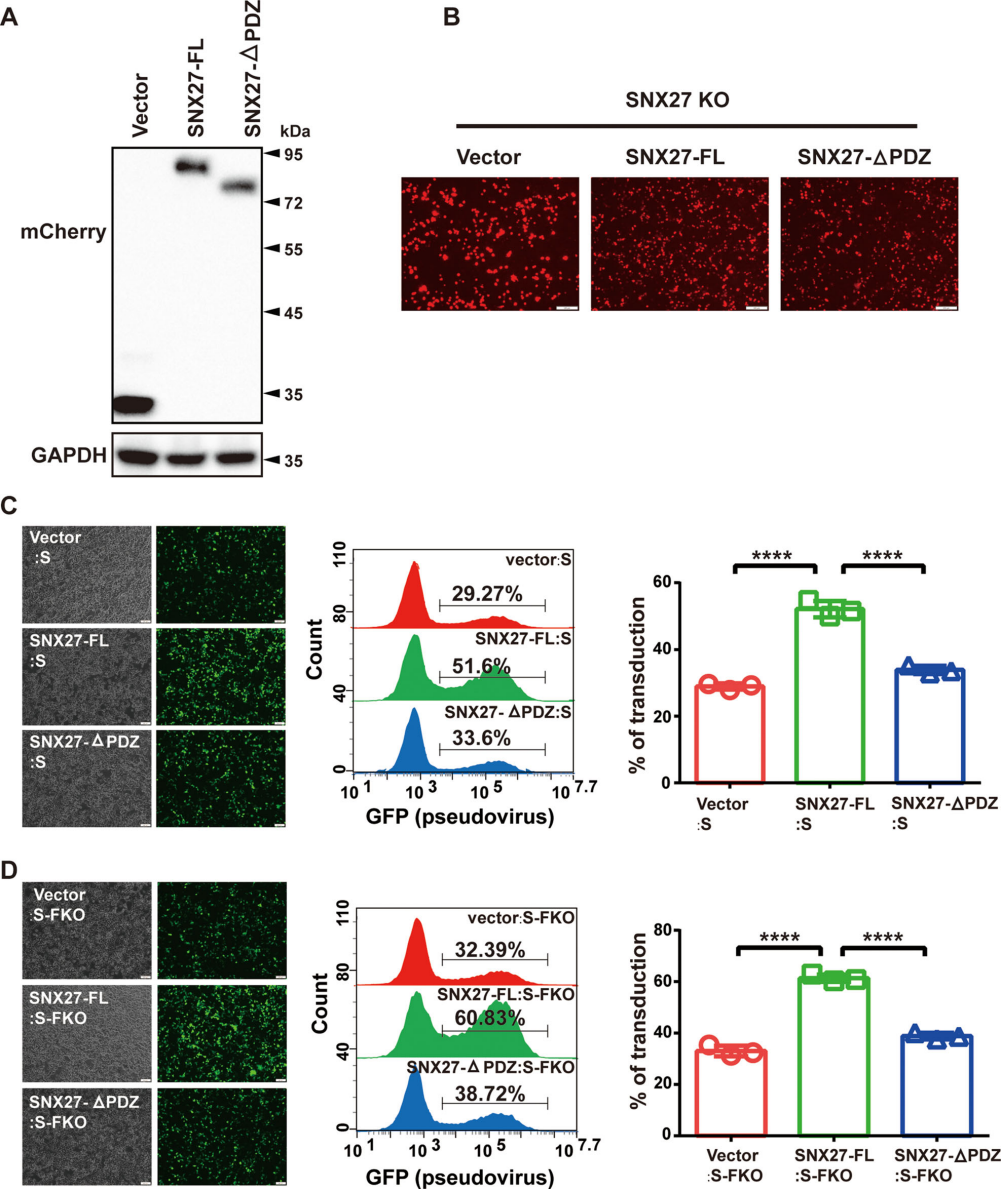
The PDZ domain of SNX27 is required to promote transduction of the S‐bearing pseudovirus. (A), (B) SNX27 KO HEK293T cells were reexpressed with empty vector, mCherry‐SNX27‐FL and mCherry‐SNX27‐△PDZ, respectively, and were subjected to immunoblotting using antibody against mCherry. (B) Representative fluorescence images of A. (C) Fluorescence and flow cytometry analysis of transduction efficiency of the S‐bearing pseudoviruses produced from cells in A and B. Left: representative fluorescence images from one experiment. Middle: representative flow cytometry results from one experiment. Right: the percent of GFP positive cells, determined by flow cytometry, is used to calculate viral transduction efficiency. Data are mean ± SD of three independent experiments. ****p < 0.001. Scale bar: 100 µm. (D) Fluorescence and flow cytometry analysis of transduction efficiency of the S‐FKO‐bearing pseudoviruses produced from cells in A and B. Left: representative fluorescence images from one experiment. Middle: representative flow cytometry results from one experiment. Right: the percent of GFP positive cells, determined by flow cytometry, is used to calculate viral transduction efficiency. Data are mean ± SD of three independent experiments. ****p < 0.001. Scale bar: 100 µm

To demonstrate the importance of PDZ domain for the production and transduction of S‐bearing pseudovirus, we re‐expressed vector, full‐length SNX27 (SNX27‐FL), or SNX27‐△PDZ in SNX27 KO cells (Figures 5A and 5B). Both immunofluorescence and flow cytometry revealed that significantly more cells were infected by S‐bearing pseudovirus produced in cells with SNX27‐FL (∼52%), relative to cells with vector (∼29%) or SNX27‐△PDZ (∼34%) (Figure 5C). Similar results were obtained for S‐FKO‐bearing pseudovirus (Figure 5D). Thus, the PDZ domain of SNX27 is critical for S‐bearing and S‐FKO‐bearing pseudovirus transduction.

### 2.4 The PDZ domain of SNX27 binds to the “MTSC” motif of S protein to promote viral transduction

**FIGURE 6:**
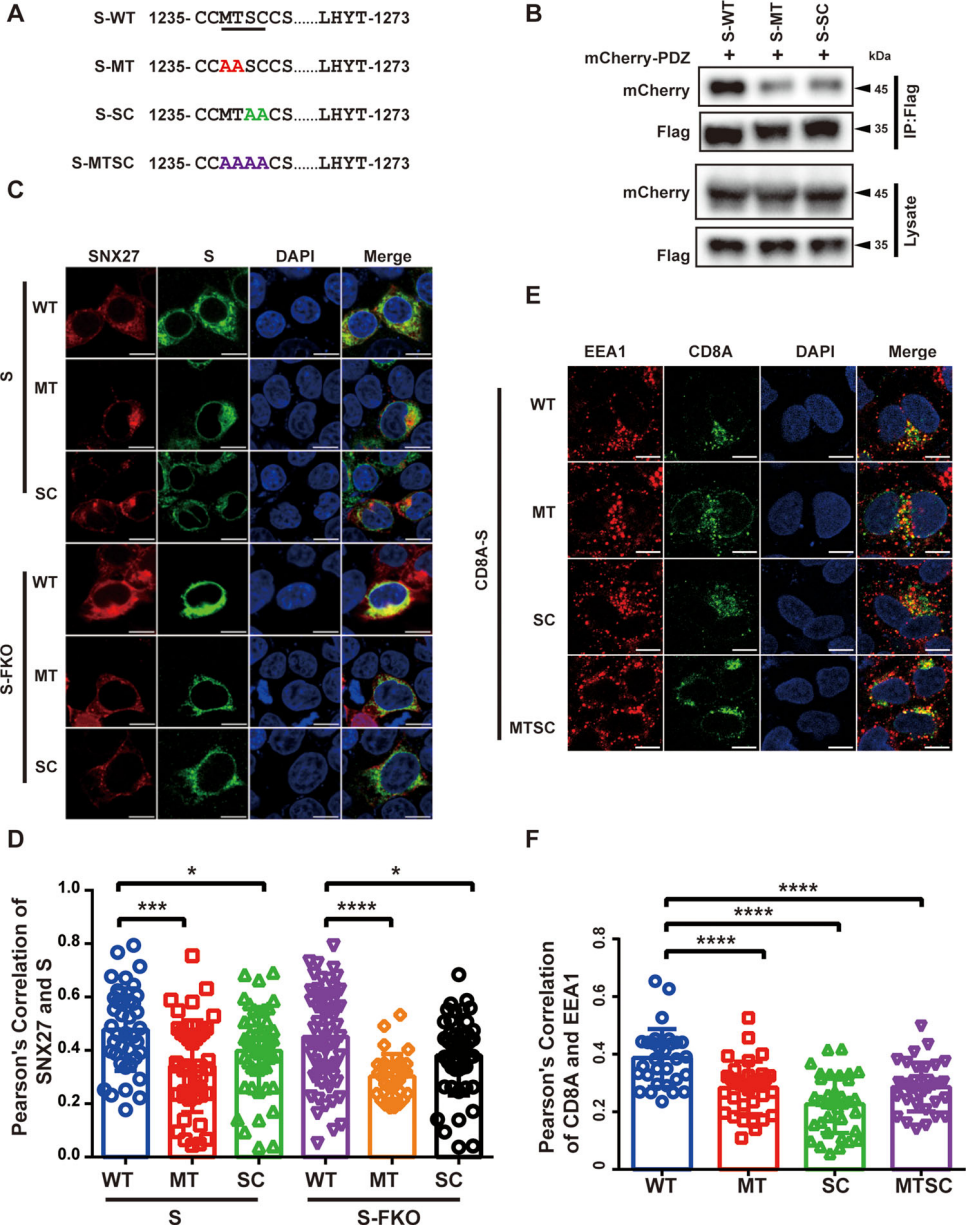
SNX27‐PDZ binds to the “MTSC” motif of S protein to promote the endocytic recycling of S protein. (A) Schematic representation of S protein wildtype and mutants. (B) HEK293T cells were cotransfected with mCherry‐SNX27‐PDZ and S‐Flag. Immunoprecipitation (IP) was performed using anti‐Flag magnetic beads, followed by immunoblotting with indicated antibodies. (C) Confocal immunofluorescence of HEK293T cells cotransfected with S‐Flag‐WT, ‐MT, ‐SC, S‐Flag‐FKO‐WT, ‐MT or ‐SC and mCherry‐ SNX27 for 24 h. S protein was stained with anti‐Flag monoclonal antibody (green), and nuclei stained with DAPI (blue). Scale bar: 10 µm. (D) Colocalization of S protein and mCherry‐SNX27 in cells in a. Pearson's coefficients of S protein and mCherry‐SNX27 were calculated using Image J. Each dot represents the value of one cell. *N* = 3 independent experiments. *p* Values were calculated using one‐way ANOVA, Tukey's multiple comparisons test. *p < 0.05; ****p < 0.0001. (E) Confocal immunofluorescence of HeLa cells transfected with CD8A‐S, CD8A‐S‐MT, CD8A‐S‐SC, and CD8A‐S‐MTSC. Cells were incubated with monoclonal anti‐CD8A antibody on ice for 30 min. Unbound antibodies were removed, and the internalization of antibody‐bound CD8A was chased in DMEM at 37˚C for 30 min. The internalized CD8A‐antibody was detected using Alexa‐488 secondary antibodies, early endosome was stained anti‐EEA1antibody (red), and nuclei was stained with DAPI (blue). Scale bar: 10 µm (left). (F) Colocalization of CD8A and EEA1 in cells in C. Pearson's coefficients of CD8A‐S and EEA1 were calculated using Image J. Each dot represents the value of one cell. *N* = 3 independent experiments. *p* Values were calculated using one‐way ANOVA, Tukey's multiple comparisons test. *p < 0.05; ****p < 0.001

Previous study has identified that the “MTSC” motif at the cytoplasmic end of S protein is required for the binding to SNX27.^22^ Consistently, our immunoprecipitation experiments showed that mutations of either MT or SC residues significantly reduced the binding of S protein to SNX27‐PDZ (Figures 6A and 6B). Less than 50% of SNX27‐PDZ was retained by either S‐MT or S‐SC, relative to S‐WT (Figure 6B). Immunofluorescence experiments also revealed that impaired binding of SNX27 to S protein also resulted in decreased colocalization of SNX27 and S protein (Figures 6C and 6D). Anti‐CD8A antibody uptake assays future demonstrated that both the double mutants (CD8A‐S‐MT or S‐SC) and the quadruple mutant (S‐MTSC) displayed reduced colocalization with EEA1 compared to S‐WT (Figures 6E and 6F). Altogether, these data suggest that SNX27 binds to the “MTSC” motif of S protein and regulates endocytic trafficking of S protein.

To investigate the importance of the “MTSC” motif for viral transition, we produced S‐WT, S‐MT or S‐SC bearing pseudovirus, and used to infect HEK293T‐ACE2 cells. The transduction efficiency of S‐WT bearing pseudovirus was nearly twice that of S‐MT or S‐SC (Figure S4A). Similarly, the S‐FKO‐WT pseudovirus was more effective to transduce HEK293T‐ACE2 cells than their mutant counterparts (Figure S4B). To further verify the importance of SNX27‐S interaction for viral transduction, we also generated S‐MT or S‐SC bearing pseudovirus in control, SNX27 KO‐1 or SNX27 KO‐2 cells, and compared with S‐WT bearing pseudovirus (Figure S5). Depletion of SNX27 led to ∼30% reduction in the transduction efficiency of S‐WT bearing pseudovirus; in contrast, the transduction efficiency of S‐MT and S‐SC bearing pseudoviruses was about same in control and SNX27 KO cells (Figure S5). Taken together, the interaction between SNX27‐PDZ and the “MTSC” motif of S protein likely promotes the surface expression of S protein and viral production.

## 3 DISCUSSION

**FIGURE 7:**
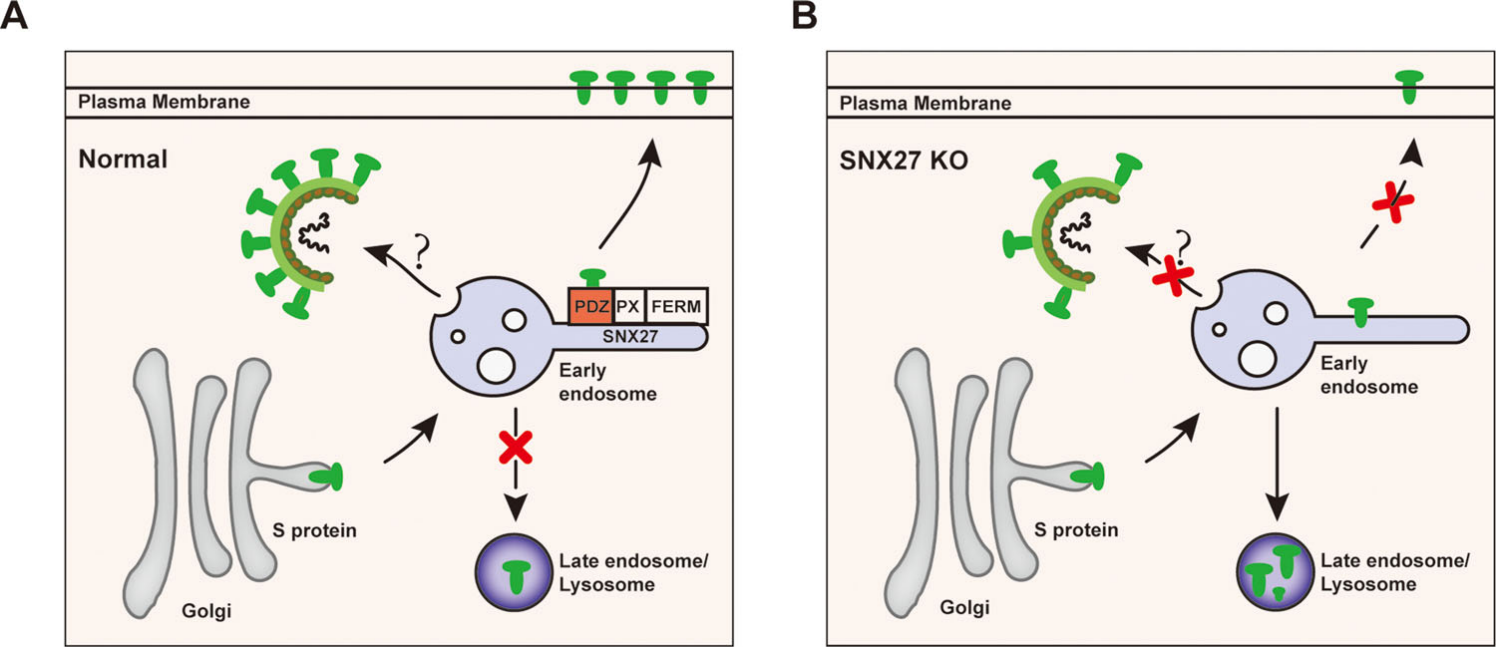
Proposed model showing SNX27 promotes intracellular trafficking of S protein and viral production. (A) SNX27, via its PDZ domain, interacts with S protein and promotes endosome‐to‐plasma membrane trafficking of S protein. SNX27 could also promote the production of SARS‐CoV‐2 virions in host cells, although the mechanism remains poorly defined. (B) Depletion of SNX27 impairs endosome‐to‐plasma membrane trafficking of S protein, leading to its lysosomal degradation

Many viruses and intracellular bacteria are known to hijack endocytic trafficking pathway during the infection.^29^, ^30^, ^31^, ^32^, ^33^, ^34^ Here, we identify a novel mechanism employed by SARS‐CoV‐2 to facilitate virion trafficking to establish virus infection. We find that SNX27 mediates endocytic trafficking of S protein and promotes its surface expression. Either abrogation of SNX27 or the “MTSC” motif of S protein, which is critical for the SNX27 binding, decreases the surface expression of S protein and viral production. Our study suggests that SNX27 and possibly other endocytic trafficking regulators, in addition to regulating surface expression of host protein ACE2,^20^, ^24^ are exploited by SARS‐CoV‐2 to complete the viral life cycle (Figure 7).

One of our most interesting discoveries is that S protein binds to the PDZ domain of SNX27, despite that it does not encompass a PDZbm. Although the PDZ domains are best known to recognize C‐terminal peptides, certain PDZ domains can bind to internal sequence “uncanonically.”^35^ For instance, the PDZ domain of human Dvl2 is able to bind a large panel of internal sequences.^36^ More interestingly, SNX27 is reported to mediate human papillomavirus (HPV) infection by directly binding to a central fragment of HPV‐16 L2 protein via its PDZ domain.^37^ Such an interaction promotes pseudovirion infection.^37^ Future studies will be necessary to address how SNX27 interacts with SARS‐CoV‐2 S protein and HPV‐16 L2 protein.

So far, no specific antiviral drug has been shown to be effective in the treatment of SARS‐CoV‐2 infection.^38^ The presence of S protein at cell surface helps to induce cell fusion and to form multinucleate syncytia.^39^, ^40^ Our study indicates that SNX27 and other endosomal trafficking components could regulate surface expression of S protein and syncytia formation by SARS‐CoV‐2. Thus, targeting endosomal trafficking pathway represents a novel approach to prevent or resolve SARS‐CoV‐2 infections. Two small molecules that target host endolysosomal pathways, Retro2 and ABMA, have been shown to protect cells against multiple toxins and pathogens.^41^, ^42^ It will be interesting to determine whether these molecules are effective to treat the emerging SARS‐CoV‐2.

## 4 MATERIALS AND METHODS

### 4.1 Cell culture and transfection

HEK293T and HeLa Cells used in this study were cultured in Dulbecco's modified Eagles medium (DMEM, Gibco), adding with 10% (v/v) fetal bovine serum (Biological Industries,BI), penicillin‐streptomycin 1% (v/v) (Hyclone) and BIOMYC‐3 Antibiotic Solution 1% (v/v) (Biological Industries, BI). Cells were maintained in a standard CO2 humidified incubator with 5% CO2 at 37˚C. HeLa cells were transfected by Lipofectamine™ 3000 Reagent (Invitrogen, L3000015) and HE293T cells were transfected using Liposomal Transfection Reagent (Yeasen, 40802ES03) according to the manufacturer's protocol.

### 4.2 Generation of CRISPR‐KO cell lines

SNX27‐KO HeLa cell lines were produced in our previous study.^17^ SNX27‐KO HEK293T cell lines were performed as previously described.^17^, ^43^ Briefly, the two guide RNAs (gRNAs) targeting SNX27 distinct genomic regions (gifts from Dr. Florian Steinberg) were, respectively, cotransfected with a plasmid encoding GFP‐tagged puromycin resistance into HEK293T cells at a ratio of 1:1. After 48 h of transfection, selective medium with 8 µg/ml puromycin dihydrochloride (Beyotime, ST551‐10) were added for 24 h. Dead cells were removed by wash with PBS (Hyclone) three times. Then the remaining cells were maintained with refresh nonselective medium every other day until they reached 80%–90% confluence. The expression of SNX27 was determined by immunoblotting

### 4.3 Immunofluorescence and confocal microscopy

Immunofluorescence staining was performed as previously described.^44^, ^45^ Briefly, cells were washed with ice‐cold PBS, fixed with 4% PFA for 10–20 min, washed with PBS twice again and permeabilized with 0.1% Triton X‐100 in PBS for 15 min. Cells were blocked with 5% BSA in PBS, and then incubated with these indicated antibodies. Antibodies that did not bind the protein of interest or that bound nonspecifically were washed away by ice‐cold PBS. Next cells were incubated with the second antibodies. Images were acquired by Olympus FV‐1000 and Olympus FV‐3000 confocal microscope and analyzed by NIH ImageJ software.

### 4.4 Surface protein internalization and recycling assays

The CD8A‐tagged constructs internalization and recycling were performed as previously described.^17^ Briefly, Control or SNX27 KO HeLa cells were transfected with plasmids encoding CD8A‐tagged constructs. After 24 h of transfection. 5 µg/ml monoclonal antihuman CD8A antibody (Invitrogen, 14‐0086‐80) was added on ice for 30 min. Redundant antibodies were washed away by with ice‐cold PBS three times. The internalization of antibody‐bound CD8A‐tagged constructs complexes chase in DMEM at 37˚C for 30 min (detection the colocalization of CD8A with EEA1) or 60 min (detection the colocalization of CD8A with LAMP1). At indicated time points, cells were taken out for immunofluorescence staining with Alexa‐488 or 546‐conjugated secondary antibodies (Jackson), and then detected by confocal microscopy.

### 4.5 Protein degradation assay

Protein degradation assay was performed as previously described.^17^, ^46^ Plasmids encoding S protein WT or mutants were transiently transfected into HEK293T cells using Liposomal Transfection Reagent (Yeasen, 40802ES03). Twenty‐four hours after transfection, ribosomal inhibitor cycloheximide (CHX, 25 µg/ml) was added into the wells at different time points. Cells were harvested at the same time, lysed with 1×loading buffer, and used for immunoblotting. Four independent experiments results were quantified.

### 4.6 S‐bearing pseudovirus production and infection

S‐bearing pseudovirus production and infection were performed as previously described.^23^ Briefly, HEK293T cells were transfected with plasmids encoding codon‐optimized SARS‐CoV‐2 spike protein with C‐terminal 18aa truncated, a pLenti‐EGFP vector (Addgene, 17448) and a gag/pol expression plasmid (Addgene, 12260) using polyethyleninime (Polysciences). Six hours posttransfection, new complete culture medium were added. Forty‐eight hours postinfection, the culture supernatants containing EGFP‐expressing pseudoviruses were harvested and centrifugation. The supernatants were filtered through 0.45 µM pore‐size filter (Millipore, SLHP033RB). Supernatants containing pseudovirus were added to HEK293T/ACE2 cells. Media was changed the following day and 48 h after infection, EGFP expression in infected cell was determined by fluorescent microscopy and flow cytometry.

### 4.7 Examination of S expression on cell membrane

Plasmids encoding S protein were transfected into wild type or SNX27 KO HEK293T cells at approximately 60% confluence in a 6‐well plate with PEI. Twenty‐four hours after transfection, the cells were harvested, stained with anti‐S primary antibody (GeneTex, GTX632604) and APC‐conjugated goat antimouse second antibody (Thermo Scientific, F542), and then subjected to flow cytometry analysis (Agilent, USA).

### 4.8 Immunoprecipitation

HEK293T cells were cotransfected with mCherry‐SNX27 and S‐Flag. Cells were then lysed into lysis buffer supplemented with 1% protease inhibitor cocktail (Bimake, B14001), phosphatase inhibitor cocktail A (Bimake, B15001) and phosphatase inhibitor cocktail B (Bimake, B15002), rotated at 4°C for 30 min. After centrifugation, the supernatant was collected. Ten percent of the whole cell lysate was used as lysate, and the rest was prepared for immunoprecipitation with anti‐Fag magnetic beads at 4°C overnight. The beads were washed six times with ice‐cold PBS, boiled for 10 min at 98°C, and analyzed by immunoblotting.

### 4.9 Statistical analysis

All cellular experiments were duplicated at least three times, and representative results are presented. Measurement data are expressed as mean ± SD and were analyzed using a one‐way ANOVA test followed by a Tukey's test in GraphPad Prism 7.0. Colocalization analysis of immunofluorescence images was performed with the software FV31S‐SW V2.4 Viewer or ImageJ. Statistical analyses were performed using one‐way ANOVA incorporated in Prism 7 (GraphPad Software). ****p < 0.001, **p < 0.01, *p < 0.05, ns: not significant.

## CONFLICTS OF INTEREST

The authors declare no conflict of interest.

## AUTHOR CONTRIBUTIONS

L.Z. performed cellular studies with assistance of J.Z., and K.Z. performed viral studies. X.Y. provided technical assistance. A.T. and D.J. supervised the project, and prepared the manuscript with input from every author.

## ETHICS APPROVAL

This project was permitted by the Independent Ethics Committee of Sichuan University. This research conforms to all the laws and ethical guidelines that apply in the country.

## Supporting information

Supporting information

## Data Availability

All data are available from the corresponding authors upon request.
