# Supplementary material for: SARS‐CoV‐2 spike protein harnesses SNX27‐mediated endocytic recycling pathway
Source: MedComm (2020). 2021 Oct 8;2(4):798–809. doi: 10.1002/mco2.92 (PMC8661858; doi:10.1002/mco2.92)
Supplement: Supplementary file 1 — Supporting information [file MCO2-2-798-s001.pdf]

SARS-CoV-2 spike protein harnesses SNX27-mediated endocytic recycling pathway

Lin Zhao[1,#], Kunhong Zhong[2,#], Jia Zhao[1,#], Xin Yong[1], Aiping Tong[2,*], Da Jia[1,*]

[1]Key Laboratory of Birth Defects and Related Diseases of Women and Children, Department of Paediatrics, West China Second University Hospital, State Key Laboratory of Biotherapy and Collaborative Innovation Center of Biotherapy, Sichuan University, Chengdu 610041, China

[2] State Key Laboratory of Biotherapy and Cancer Center, West China Hospital, West China Medical School, Sichuan University, Chengdu 610041, China

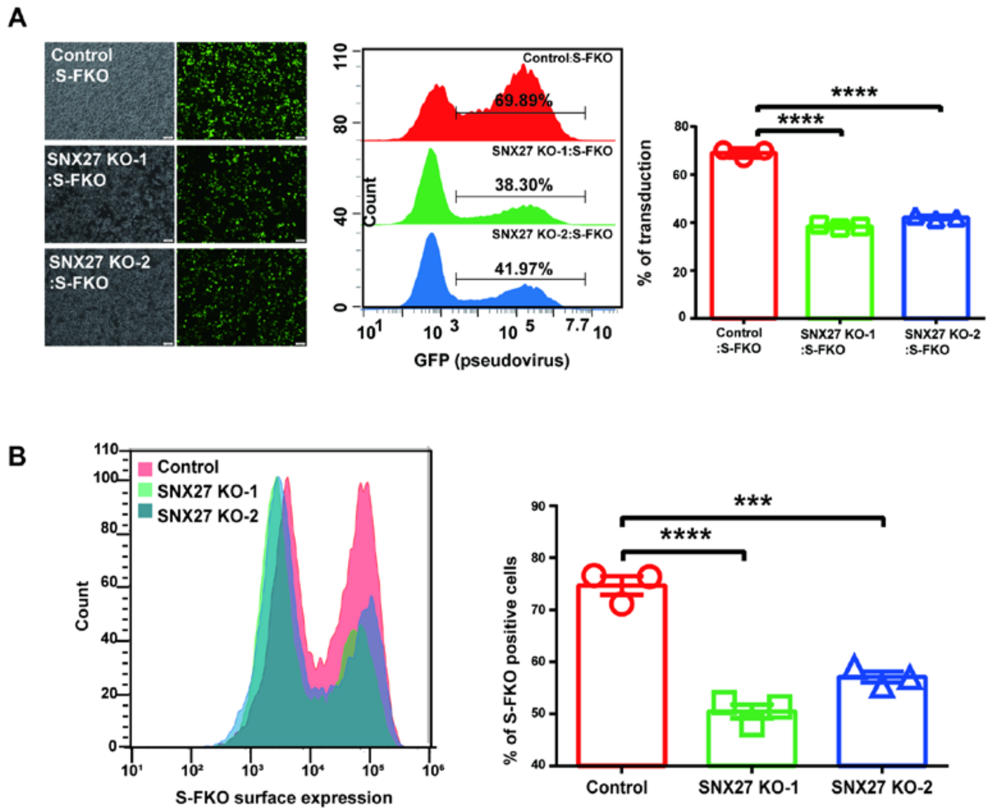

Fig. (S1). SNX27 promotes cell surface expression of S-FKO protein and transduction efficiency of the S-FKO-bearing pseudovirus

(A) Fluorescence and flow cytometry analysis of transduction efficiency of the S-FKO-bearing pseudoviruses produced from cells in Fig 1.B. Left: representative fluorescence images from one experiment. Middle: representative flow cytometry histograms from one experiment. Right: the percent of GFP positive cells, determined by flow cytometry, is used to calculate viral transduction efficiency. Data are mean ±SD of three independent experiments. ****p < 0.0001. Scale bar:100 µm.

(B) Determination of S-FKO protein cell surface expression by flow cytometry. HEK293T cells were transiently transfected with plasmids encoding full-length S-FKO. Twenty-four hours after transduction, cells were collected and stained with anti-S antibody (which recognizes extracellular

domain of S protein), followed by flow cytometer analysis. Left: flow cytometry results from one representative experiment. Right: percent of S-positive cells. Statistic data represent the results from n = 3 independent experiments and are expressed as mean ± SD. ****p < 0.0001. Scale bar:100 μm.

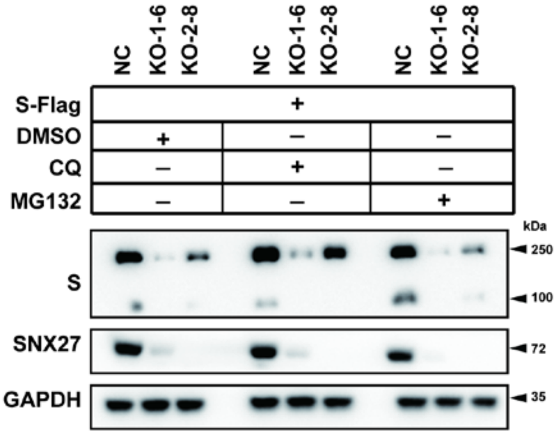

Fig. (S2). Lysosomal inhibitor Chloroquine (CQ) partially restores the degradation of S protein resulting from SNX27 knockout.

Control and two clonal SNX27-KO cell lines were transiently transfected with plasmid encoding S protein. 24h after transfection, cells were treated with DMSO, Chloroquine (CQ, 25 μM) or MG132 (10 μM) for 8h, respectively. Cells were collected for western blotting.

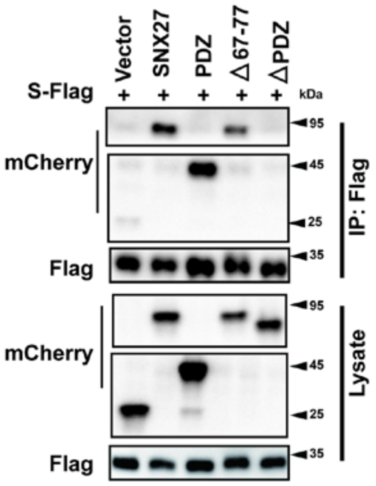

Fig. (S3). The PDZ domain alone of SNX can bind the S protein in a retromer-independent manner.

HEK293T cells were co-transfected with mCherry-SNX27 and S-Flag for 24 h. Control cells (vector) were transfected with empty vector. One tenth of the cell lysate was prepared as lysate, and the rest was used for immunoprecipitation with anti-Flag magnetic beads, followed by immunoblotting. Representative images from one of three independent experiments are shown.

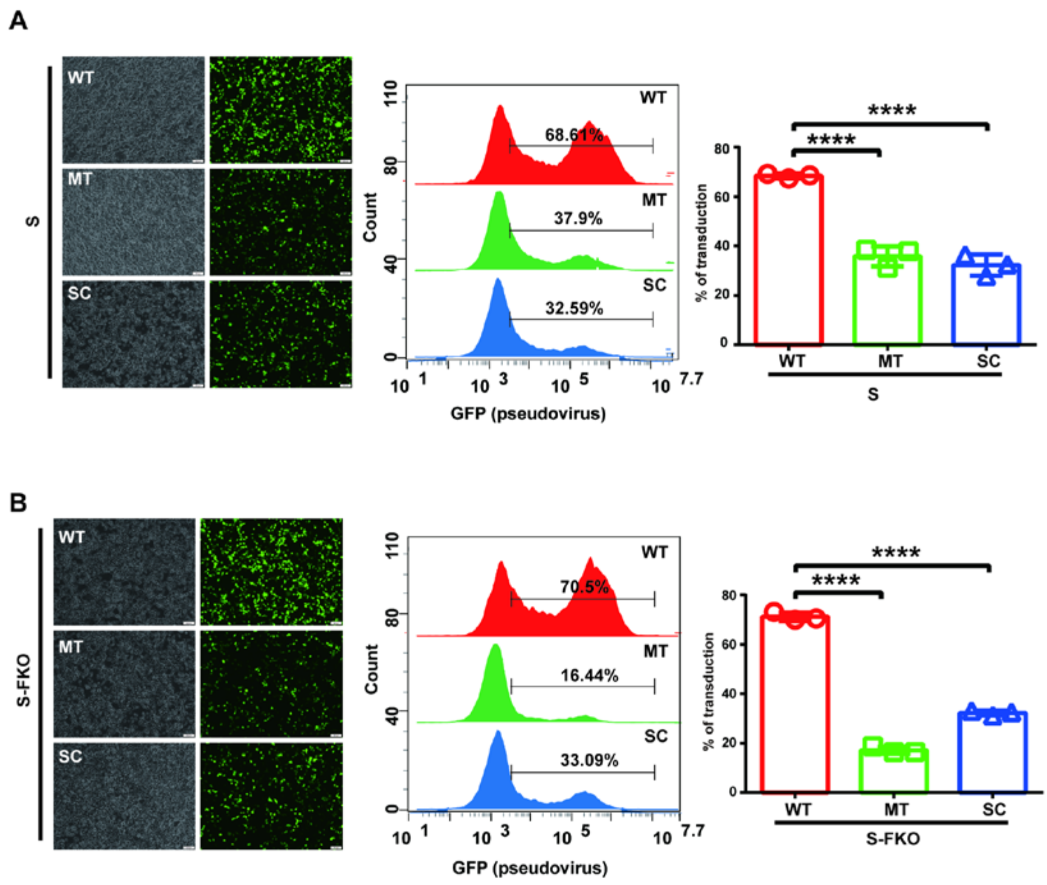

Fig. (S4). The "MTSC" motif in S protein is critical for S-bearing pseudovirus transduction

(A) Fluorescence and flow cytometry analysis of transduction efficiency of the S-bearing pseudoviruses. Left: representative fluorescence images from one experiment. Middle: representative flow cytometry histograms from one experiment. Right: the percent of GFP positive cells, determined by flow cytometry, is used to calculate viral transduction efficiency. Data are mean ± SD of three independent experiments. **** $p < 0.001$. Scale bar: 100 µm.

(B) Fluorescence and flow cytometry analysis of transduction efficiency of the S-FKO-bearing

pseudoviruses. Left: representative fluorescence images from one experiment. Middle: representative flow cytometry histograms from one experiment. Right: the percent of GFP positive cells, determined by flow cytometry, is used to calculate viral transduction efficiency. Data are mean ± SD of three independent experiments. **** $p < 0.001$. Scale bar: 100 μm.

A

S-WT			S-MT			S-SC		
Control	SNX27 KO-1	SNX27 KO-2	Control	SNX27 KO-1	SNX27 KO-2	Control	SNX27 KO-1	SNX27 KO-2
64.8	42.01	47.31	41.23	36.56	40.82	31.05	33.32	34.16
67	42.88	47.89	41.5	35.86	41.69	29.71	32.78	35.38
64.36	41.88	46.58	37.68	37.09	39.88	33.54	33.76	34.09

B

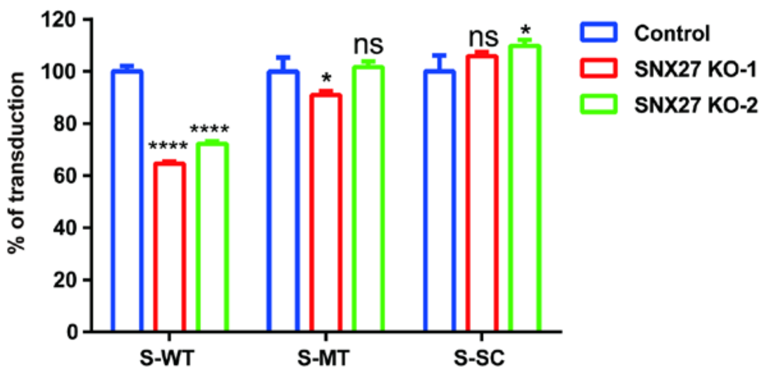

Fig. (S5). The SNX27-S interaction promotes transduction of S-bearing pseudovirus.

(A-B) Flow cytometry analysis of transduction efficiency of the S-bearing pseudoviruses. S-WT, S-MT and S-SC bearing pseudoviruses were produced form control, SNX27 KO-1 and SNX27 KO-2 cells, and used to transduce HEK293T-ACE2 cells. Flow cytometry was used to determine the transduction efficiency. A: percent of GFP-positive HEK293T-ACE2 cells. B: Quantification of transduction efficiency of pseudoviruses produced from different cells. % of transduction is calculated by transduction efficiency of pseudoviruses produced from control or SNX27 KO cells

divided by that from control cells, as shown in A. N = 3 independent experiments. P values were calculated using one-way ANOVA, Tukey's multiple comparisons test. Ns: no significant difference. * $p < 0.05$; **** $p < 0.001$, ns: not significant.
